# P-2169. Establishment of multiplex PCR method for nearly whole-genome sequencing of human parainfluenza/rubula virus 4 from a clinical sample

**DOI:** 10.1093/ofid/ofae631.2323

**Published:** 2025-01-29

**Authors:** Yusuke Sayama, Masahiro Sakamoto, Michiko Okamoto, Mayuko Saito, Raita Tamaki, Mariko Saito-Obata, Christina Dahlia Joboco, Reynaldo Frederick Negosa Quicho, Socorro P Lupisan, Hitoshi Oshitani

**Affiliations:** Tohoku University Graduate School of Medicine, Sendai, Miyagi, Japan; Tohoku University Graduate School of Medicine, Sendai, Miyagi, Japan; Tohoku University Graduate School of Medicine, Sendai, Miyagi, Japan; Tohoku University Graduate School of Medicine, Sendai, Miyagi, Japan; Nagasaki University, Nagasaki, Nagasaki, Japan; Tohoku University Graduate School of Medicine, Sendai, Miyagi, Japan; Biliran Provincial Hospital, Naval, Biliran, Philippines; Ospital ng Palawan, Puerto Princesa City, Palawan, Philippines; Research Institute for Tropical Medicine, Muntinlupa City, National Capital Region, Philippines; Tohoku University Graduate School of Medicine, Sendai, Miyagi, Japan

## Abstract

**Background:**

Human parainfluenza/rubula virus 4 (HPIV4) has two subtypes (4a/4b). HPIV4 infection is usually mild but can be severe in young children, older adults, and immunocompromised individuals. However, sequence information on HPIV4 is limited worldwide, including in tropical areas. Whole-genome sequencing is useful for the molecular epidemiological analysis of virus introduction and transmission routes. Thus, this study aimed to establish nearly whole-genome sequencing of HPIV4 subtypes using multiplex PCR (mPCR) methods and to identify genetic characterization in the Philippines.

**Methods:**

We collected 29 (4a; n=17, 4b; n=12) HPIV4-positive samples from children (< 5 years old) with severe pneumonia at three hospitals (Leyte, Biliran, and Palawan) in the Philippines from 2012 to 2019. HPIV4 positive samples showed cycle threshold (Ct) values ranging from 21.1 to 36.9. An online pipeline tool (Primal Scheme) was used to design mPCR primer panels for HPIV4 subtype-specific viruses to cover the whole genome. For the sequencing experiment, viral RNA was purified from clinical samples and synthesized cDNA. cDNA was amplified using mPCR with each primer panel. The PCR amplicon was library preparation with a tag for the Illumina sequencing platform. Data of whole-genome sequencing was obtained using a CLC genomic workbench.

**Results:**

All of the 29 HPIV4-positive samples were amplified by mPCR for whole-genome sequencing. The whole-genome sequencing results for HPIV4a showed that the length of consensus sequences generated at a coverage depth of 30X ranged from 85% to 99%. The sample with the lowest coverage (85%) had a Ct value 28.9. The results for HPIV4b showed that at a coverage depth of 30X, the length of consensus sequences ranged from 97% to 99%. The Ct values of the samples that showed 97% coverage were 22.6 and 30.4. Although some HPIV4 strains detected in the Philippines belonged to the same clade, others were in different clades. These clades included strains detected in various areas, such as Asia and North America.

**Conclusion:**

This study established that the HPIV4 subtype-specific primer panels allow users to obtain the whole genome of HPIV4 directly from clinical samples. This method provides a universal, rapid, and effective tool for genomics surveillance.

**Disclosures:**

All Authors: No reported disclosures

